# Clinical Proteomics, Quo Vadis?

**DOI:** 10.1002/pmic.202400346

**Published:** 2025-02-09

**Authors:** Harald Mischak, Joost P. Schanstra, Antonia Vlahou, Joachim Beige

**Affiliations:** ^1^ Mosaiques Diagnostics GmbH Hannover Germany; ^2^ Institute of Cardiovascular and Metabolic Disease U1297 Institut National de la Santé et de la Recherche Médicale Toulouse France; ^3^ Université de Toulouse Toulouse France; ^4^ Center of Systems Biology Biomedical Research Foundation of the Academy of Athens Athens Greece; ^5^ Martin‐Luther‐University Halle‐Wittenberg Halle (Saale) Germany; ^6^ Kuratorium for Dialysis and Transplantation Leipzig Germany

**Keywords:** clinical proteomics, clinical study, implementation, patient benefit

## Abstract

The field of clinical proteomics has seen enormous growth in the past 20 years, with over 40,000 scientific manuscripts published to date. At the same time, actual clinical application of the reported findings is obviously scarce. In this viewpoint article, we discuss the key issues that may be responsible for this apparent lack of success. We conclude that success must not be assessed based on the number of publications, but via the impact on patient management and treatment. We proceed with specific suggestions for potential solutions, which include keeping a strict focus on potential patient benefit. We hope this article can help shape the field, so it can in fact deliver on its realistic promise to bring significant improvement in management and care to patients.

## 1 Introduction

The field of clinical proteomics, which emerged over 25 years ago, held transformative promise for medical science, aiming towards revolutionising patient care through precision in diagnosis, prognosis and treatment [1]. The foundational idea is that proteins through their critical function as enzymes catalyse all biological reactions, thus regulating essentially all aspects of biological life. Controlling of these activities is only to a small degree determined by gene expression (transcription and translation) to regulate the protein quantity. Further levels of control, specifically regulation of the protein activity or function are generally more relevant in controlling cell function. Consequently, changes in protein levels, modifications and interactions reflect—and in general cause—noncommunicable disease. Proteomic research aimed to identify biomarkers to assess disease onset and progression, decode disease mechanisms and discover drug targets with precision [2]. However, a retrospective look at clinical proteomics reveals that, despite substantial scientific output, its direct impact on patient outcomes has very much fallen short of initial expectations. A Medline search for ‘clinical proteom*’ retrieves well over 40,000 publications to date, with more than 16,000 of these also included when using the term ‘biomarker’. At the same time, at best a handful of examples for actual clinical application can be found today, and none of these applied in wide use. This prompts a critical examination of both obstacles and accomplishments within the field and defining potential paths forward that could better fulfil its early promises, which still hold true today.

## 2 Technical Limitations in Clinical Proteomics

**FIGURE 1:**
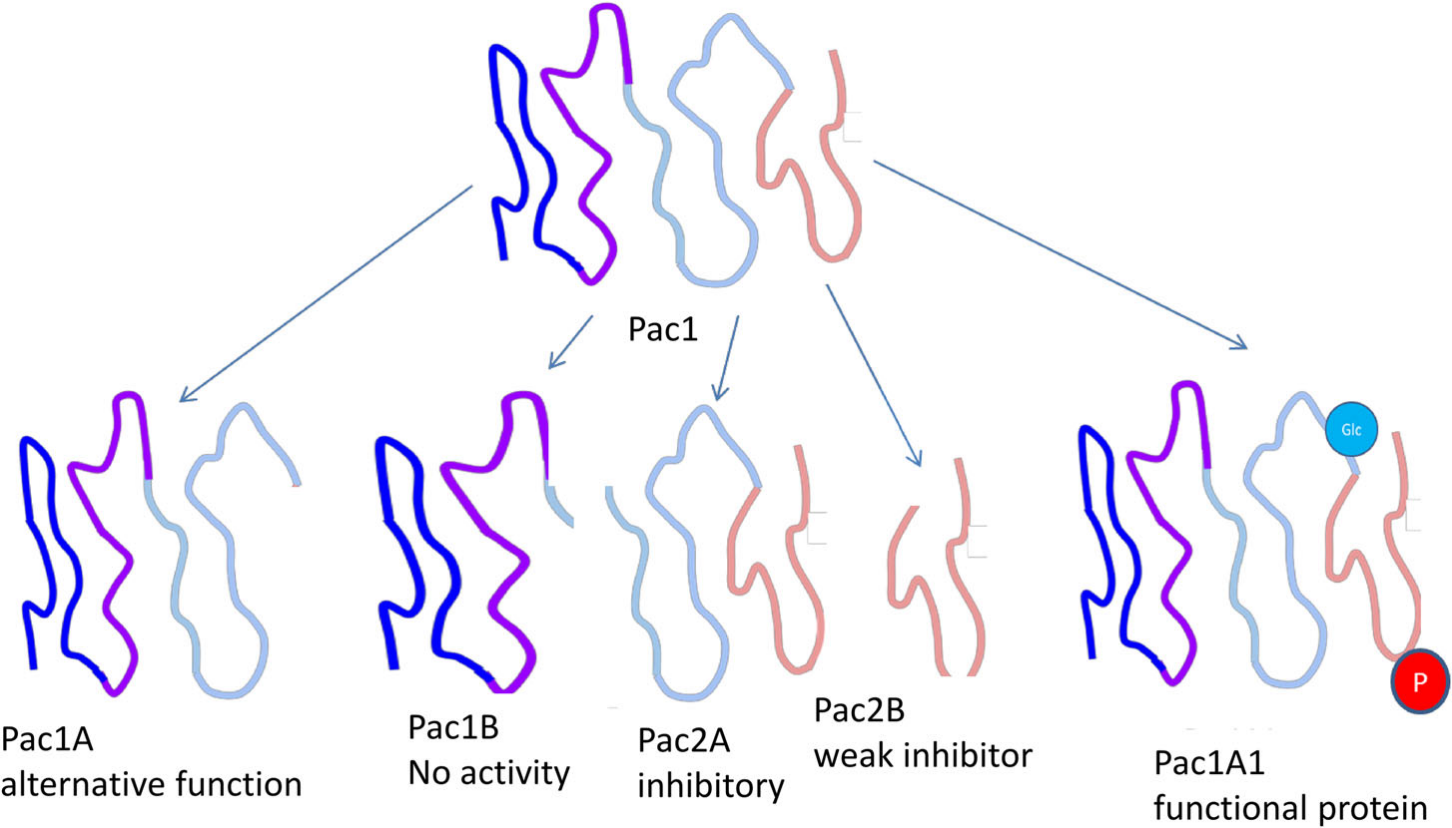
Biological functions of a hypothetical protein, Pac1, of which differently modified variants with different biological functions exist. The (hypothetical) protein Pac1 has little biological activity as a native polypeptide chain, but is converted into its active form, Pac1A1, by phosphorylation (red) and glycosylation (blue). The protein can also be specifically cleaved by proteases into Pac1A, which fulfils an alternative biological function, Pac1B which has no known function, and Pac2A and B, which inhibit the activity of Pac1A1.

At a fundamental level, proteins present analytical complexities and challenges that are unique in comparison to nucleic acids. Proteins are generated from the combinations of 20 different amino acids and are frequently modified after translation through processes like phosphorylation, glycosylation or proteolysis, which, among others, regulate their function, and practically complicate their detection (for a recent overview and review, see [3] Figure 1). Unlike nucleic acids, proteins cannot be amplified, impacting on the detection sensitivity of proteomic analyses. Furthermore, the proteome's complexity is vastly greater than that of the genome or transcriptome due to the huge dynamic range and the wide range of possible post‐translational modifications, the latter are likely responsible for a substantial portion of the observed lack of identification of more than half of the peptides in a sample when analysed by mass spectrometry [4]. Other issues preventing identification are, for example, alternative splicing or amino acid substitutions [5]. Linked to the above, the abundance of a specific protein is frequently low and often undetectable. As a result, missing (or zero abundance) values are often recorded. Multiple approaches have been described for imputation of missing values (e.g. [6, 7]); however, these missing values introduce further variability in the proteomics datasets, making comprehensive proteomic analyses even more difficult and resource‐intensive, particularly when applied to large patient cohorts. Given this complexity, clinical proteomics struggles, in fact fails to efficiently directly compete with the more straightforward and often more reproducible approaches of genomics and transcriptomics. Although the correlation between transcript and protein present has been found on average moderate [8, 9], overall it is still valid to assume that generally a significant increase in a transcript will also result in an increase at the ‘naïve’ protein level, not taking post‐translational modifications into account. As a result, it seems advisable to concentrate efforts towards areas where nucleic acid‐based assessment is not applicable like: (i) analysis of body fluids where the information on specific proteins and peptides cannot be assessed at the genome/transcriptome levels (the proteins and peptides are generally expressed at a distant location, not from the cells contained in the respective body fluid sample), or (ii) study of post‐translational modifications, including proteolytic processing. These changes, many of which are also the consequence of environmental impact, can generally not be implied from genetic information, but are essential for most proteins, especially those involved in signalling, in determining the biological function.

## 3 Platform Comparability and the Challenge of Standardisation

Another significant hurdle in clinical proteomics is the lack of comparability between analytical platforms, which hinders reproducibility and reliability in complex biomarker research or sometimes even on the level of single protein measurement. With proteomic technologies assessing highly complex samples, each technology with distinct methodologies, sensitivities and detection capabilities, comparing results between clinical studies that recruited highly varying individuals as well becomes challenging. Using the same sample and investigating the same protein, significant correlation can often not be detected when comparing different platforms (e.g. [10, 11]). Issues such as errors in sequence assignments, failure of identifying post‐translational modifications and differences in platform characteristics (e.g., antibodies vs. aptamers) contribute to this variability. Without standardised protocols and cross‐platform validation, proteomic studies face barriers in reliably translating findings into clinical applications.

## 4 Biological Variability Compounding Technical Challenges

Beyond technical variability, (clinical) proteomics must cope with biological variability, which introduces additional complexity into patient‐oriented studies. Protein concentrations in biological samples vary not only due to inherent biological diversity but also due to factors such as age, sex [12], comorbidities [13], circadian rhythms, disease cycles and lifestyle variables like diet [14], exercise [15] or environmental impact [16]. These differences necessitate large sample sizes and carefully controlled study designs to detect proteomic alterations specific to a given clinical condition. The difficulty of obtaining in a reproducible way biological samples (protein abundance may be influenced by additional factors like the time and conditions of collection) further complicates the ability to draw robust conclusions, especially based on a small sample. It appears obvious that rigorous statistical testing is essential to account for these variabilities. However, such rigorous testing is frequently not implemented, largely due to limited sample size (and consequently insufficient power), ultimately leading to the reporting of findings at a low level of confidence that lacks clinical applicability.

## 5 Clinical Translation Challenges: From Discovery to Real‐World Application

**FIGURE 2:**
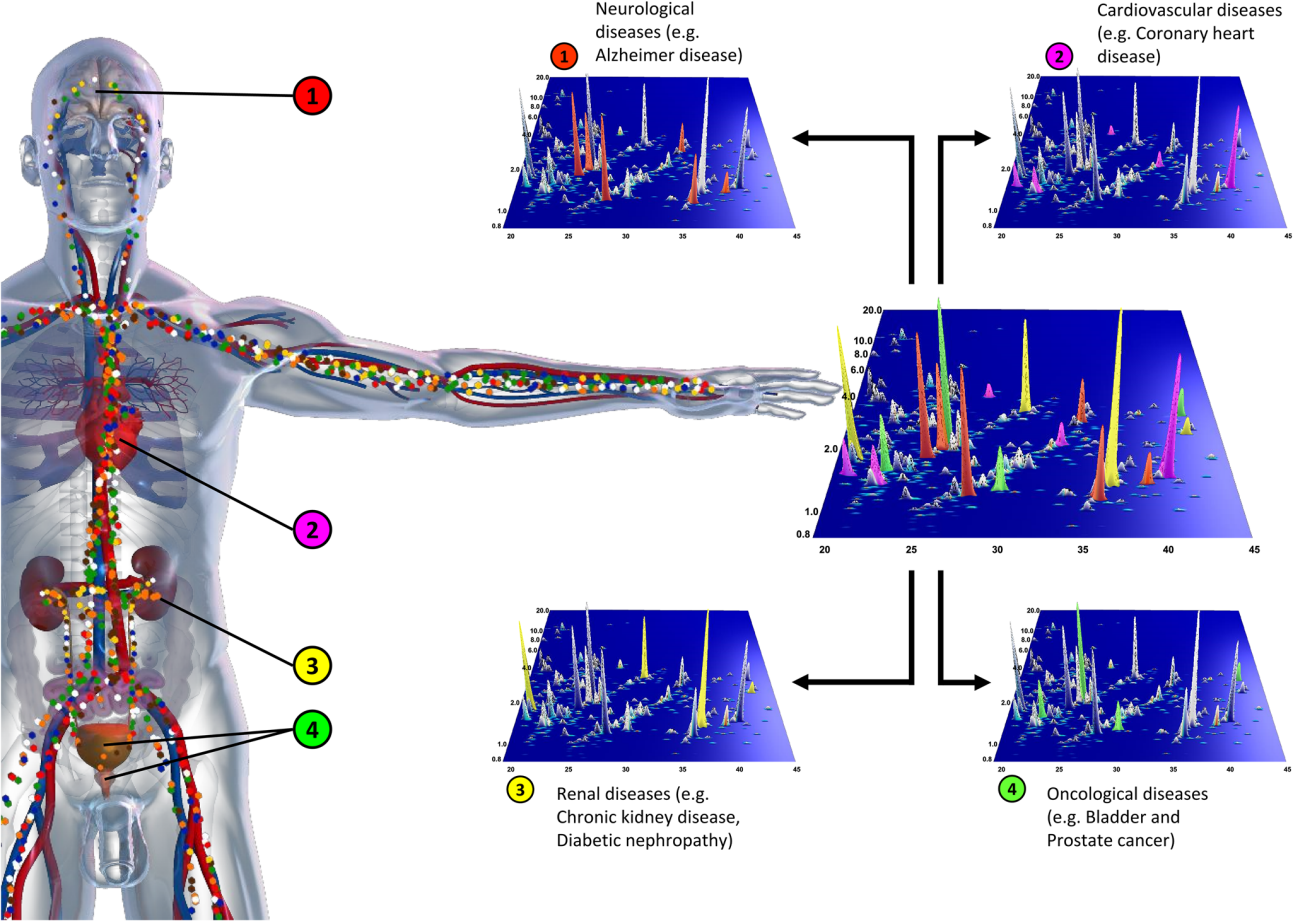
From a given sample, the proteome (multiple proteins, typically >1000) is qualitatively and quantitatively analysed. The investigation is not limited to known single biomarkers but contains information on thousands of peptides and proteins. As a result, novel biomarkers and disease models (based on the combination of biomarkers) can be identified. Linking data to the medical records of each patient, also from previous studies, allows assessing multiple individual disease profiles for precise molecular phenotyping and indication of optimal therapy. Adapted from [31].

The goal of clinical proteomics was and is improvement of patient care (Figure 2). To efficiently cope with the multiple hurdles along this path [17, 18], the original vision for clinical proteomics involved a close partnership between academic research and industry, where novel biomarkers and drug targets identified by scientists in academia would seamlessly transition to industry for development into clinical applications. Yet, this transition has proven to be more challenging than anticipated, due to, among others, issues of insufficient statistical power, lack of or poor quality validation studies in larger patient cohorts, limited clinical context or utility of many of the findings in reference to standard of care, and a lack of knowledge of regulatory requirements [18, 19]. We observed an inflation of publications on (potential) biomarkers and therapeutic targets, frequently more driven by the need to publish (or perish), rather than by solid clinical and scientific rationale. Often, the utility of discovered biomarkers or targets was and is unclear, as there may be insufficient evidence demonstrating their efficacy or added value in a real‐world setting, especially when compared with the available standard approaches. Even traditional laboratory single markers like troponin or albuminuria obtain an additional and growing part of their clinical information from a larger background and combination with related covariables like excretory kidney function, stage and origin of infection, etc. [20]. Despite being single protein markers, these traditional measures are being increasingly included in more complex multimarker signatures including these single biomarkers and clinical parameters (e.g., the kidney failure risk equation [21]), attenuating advantages of innovative (proteomic) pattern markers. For meaningful translation into patient care, proteomic discoveries must be framed within a credible, clinically relevant context, with studies designed to show clear, in most applications additive, potential patient benefits when applying the biomarker for the specific context‐of‐use. In practice, this means that, for example, a study design presenting comparisons of participants with severe disease state to healthy individuals is out of any clinical context. The same holds true for potential therapeutic targets: most of the studies are underpowered and the evidence presented for the respective potential therapeutic target is too weak to justify investing into a (pre)clinical development program. Again, many studies lack meaningful comparisons with currently available therapies to demonstrate synergistic effects or potential added benefits for the patient.

## 6 Ethical, Regulatory and Data Privacy Hurdles

The ethical and regulatory landscape adds another layer of complexity and obstacles. Ethical standards and data privacy regulations aim to protect patients and subjects included in the studies. Although this is a noble and well‐accepted goal, the road towards fulfilling the formal requirements is increasingly complicated, time‐consuming and costly [22]. Different legislation and interpretation of the ethical and data privacy regulation between different countries and the frequent vagueness on what information is considered sensitive, often delay or even entirely prevent the advancement of promising discoveries. The mere establishment of data and sample sharing agreements and ethics approval in a multi‐national project by now takes more than one, often several years, in part due to different laws in the different countries. In addition, as a result of multiple stakeholders, lawyers and complicated legal processes being involved, this process also generates substantial costs while not contributing to the research or clinical project goals. The rigor of regulatory requirements appears to be one (among others) cause for the substantial reluctance to initiate investigator‐initiated trials [23], which would be crucial for, in fact, any type of clinical research. Although ethical and data privacy considerations are relevant in general, and also for omics data, these considerations must not prevent research and in this way indirectly negatively affect patient's wellbeing, by preventing the development of better treatment. Addressing these barriers, without compromising patient safety or ethical standards, but at the same time reducing the bureaucratic and formal hurdles, is essential to enable proteomics research with real clinical potential. Although these issues are not limited to clinical proteomics, the field (as many others) is negatively impacted by them. It seems to be high time to openly discuss about the restrictions imposed by the different legislators and regulatory bodies on developing potential clinical advancements (e.g., novel biomarkers or drugs), especially with respect to the generally proclaimed aim: to serve and protect the people. By now the applicable rules and regulations appear to pose the risk of accomplishing the opposite, bringing indirect harm, by unintentionally, yet highly effectively preventing the development of beneficial solutions in patient management.

## 7 Towards a More Impactful Clinical Proteomics

To advance clinical proteomics in a meaningful way, the field must address several core challenges, as abovementioned, among others improving technical capabilities, standardising methodologies, incorporating rigorous statistical testing, and compliance with ethical, regulatory and legal requirements. Strategic efforts, like establishing cross‐platform standardisation protocols and fostering partnerships that prioritise clinical relevance, can support overcoming current barriers. Ultimately, the future success of clinical proteomics hinges on its ability to fulfil its initial promise—to deliver measurable improvements in patient outcomes through personalised diagnostics and therapies.

## 8 Specific Suggestions

### 8.1 Define Patient Benefit

**FIGURE 3:**
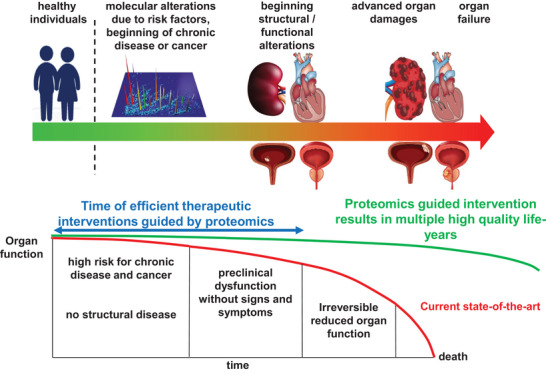
Graphic depiction of the current situation and opportunities for improvement in patient management and potential benefits of applying molecular biomarkers in the context of chronic disease. Currently, chronic diseases are typically detected based on loss of organ function, which generally cannot be recovered. Molecular biomarkers should enable early detection based on molecular changes that lead to organ dysfunction, guiding targeted intervention before irreversible organ damage occurs. Adjusted from [32].

As a first step, any clinical proteomics study should be based on the ultimate aim: towards a distinct patient benefit that should be well defined and based on the available resources and current state of the art. For example, a biomarker for the presence of advanced loss of excretory kidney function (and for kidney disease) does not appear to be of any value as (cheap) biomarkers (i.e., serum creatinine and glomerular filtration rate) for this context exist anyway. However, if a biomarker adds disease information, for example, enabling detection and earlier treatment initiation (as indicated in Figure 3), informing on tissue inflammatory response or the particular spatial localisation of the disease origin, or can be used to predict treatment response and to guide intervention [24], then it would be of value in a patient‐oriented approach (for more details in the context of kidney disease, see a recent article [20]). To define patient benefit, it is obviously essential to first define the specific context‐of‐use. It is important to consider that in general, biomarkers for the specific context‐of‐use already exist. Therefore, any new biomarker to be developed must demonstrate an added value over the current state‐of‐the‐art (or standard of care).

### 8.2 Explore Available Datasets

Typically, omics datasets (e.g., transcriptomics, proteomics and metabolomics) in the context of the targeted disease are available, but may not be thoroughly explored for the specific context‐of‐use intended. These datasets should first be investigated to support the planned study. Consequently, any clinical proteomics study should be started with a thorough search of the literature, with special emphasis on identifying available datasets. In reality, the raw datasets that are the basis of published reports unfortunately frequently turn out to not be available, even references to accession numbers in public repositories are incorrect: despite the promise in the publication, the repository does not seem to exist, the respective omics datasets cannot be found, or do not match with the clinical groups to allow meaningful comparisons [25]. However, contacting the corresponding authors responsible, if necessary including the journal editor in the communication, often is of help and the data are shared, as part of open science practices that in general is a prerequisite according to the journal policies. Based on these datasets first hypotheses and experimental approaches can be planned, with the major benefit to avoid merely repeating previously published approaches and also costly data generation to acquire profiles that already exist. Our previous study on the prediction of severe course of COVID‐19 may serve as an example. The study has an obvious patient‐relevant goal: to guide treatment aiming towards preventing severe/lethal course of COVID‐19. After reviewing the literature, a pilot study was initiated to assess if biomarkers predicting disease course may exist and to inform about the sample size requirements for a full study [26]. Funding for the full study, based on the pilot results, was attracted, and the full study was performed [27], with an interim analysis being used for the registration of the COV‐50 in vitro diagnostic test [28], which was and is available for affected patients.

### 8.3 Power Calculation Based on Preliminary or Publicly Available Data

Considering that the majority of clinical proteomics studies suffer from a lack of power, realistic estimation of the number of datasets required to reach the respective goal, to identify significant changes in the respective context of use, is obviously essential before the study initiation [29]. Next to thorough investigation of the literature, realistic power calculations, ideally based on available (preliminary) data appear a prerequisite for a successful clinical proteomics project.

### 8.4 Technical Issues Being Fully Addressed

As indicated in multiple publications, standard operating procedures (SOPs) for sampling (including, e.g., time of sampling, possible prerequisites like fasting), storage, sample preparation, sample analysis and data evaluation must all be in place. In addition, the variability and performance of the different steps must be defined and constantly assessed, ideally with a relevant standard sample. For example, if the urine proteome is to be investigated, then a sufficient amount of a standard urine sample should be available and used to ensure constant data quality (within the predefined limits that are based on the platform performance and repeatability) [30]. Overall, rigorous quality control must be implemented at every stage of the process to detect and address any deviations early, thereby ensuring the integrity and reliability of the overall workflow.

### 8.5 Strategic, Implementation‐Focused Directions by Funders

Research funding is a key driver in determining the trajectory of any research, including clinical proteomics. Funders have the power to incentivise a focus on clinically impactful studies by requiring clear, patient‐oriented objectives and a realistic implementation strategy. Many current studies, particularly in early discovery phases, lack a credible framework for translating findings into clinical practice. Without a realistic path to patient benefit, the research will almost certainly fall short of its potential. Therefore, funders should prioritise, in fact only consider for funding projects that provide a detailed, credible and realistic roadmap to clinical application and impact, which could include evidence of interest from industry partners or other stakeholders, to encourage a higher likelihood of successful translation. At the same time, driven by the exponential growth of regulatory hurdles and costs, the path towards implementation may become too costly for some funding bodies. In fact, the vast majority of public funding currently available is limited to amounts insufficient to enable performing the required clinical studies, which typically would require amounts in the range of 10 Mio US$ or € or higher [23]. Although there is no easy solution for this problem, aligning the project with the patient (who ultimately may benefit) interests and lobbying together with them for the needed public funding may be a successful strategy for a way forward.

### 8.6 Plan for Implementation

The key players in this area have very different interests. For the actual beneficiaries, the patients, the interest is quite evident: to improve their health, ideally achieve a complete recovery and experience 100% quality of life. However, patients typically do not decide on the application of certain diagnostic tools and/or drugs, such decisions are made by the physicians, often considering financial implications directed by the payers, the health insurance. Already this conflict results in developments that may appear even absurd: for example, in Germany the public health systems in fact rewards physicians for denying diagnostic tests with the ‘Wirtschaftlichkeitsbonus’ (https://www.virchowbund.de/praxisaerzte‐blog/laborbonus‐im‐ebm‐so‐erhalten‐sie‐den‐wirtschaftlichkeitsbonus). Although patients may want management/treatment optimal for their health, physicians and payers may rather want management/treatment optimal for their revenue. Last, but not least, the pharmaceutical industry has its own interest, dictated also by the requirement every company faces: to generate financial profit (as otherwise obviously the company will ultimately end up bankrupt). Development of a drug that cannot be patent protected (e.g., as a result of previous publications or repurposing strategies) and therefore is not expected to ever become profitable will not find interest from pharmaceutical companies, even if the drug shows the potential of being highly beneficial for patients. As also the public funding for such cases is typically not available (often as a result of not representing a credible business case, not being expected to be profitable), the only valid option seems to be development jointly with the patient (organisations), hopefully supported by charities.

As evident from the above, implementation is certainly not straightforward and goes far beyond scientific and clinical considerations, requires addressing of numerus nonscientific and nonclinical challenges. However, it is essential that these are being fully and realistically addressed, as otherwise the entire effort has a very good chance of failing to translate into meaningful impact or practical application, despite excellent clinical results.

## 9 Conclusion

We must keep in mind that the main, in fact, the only aim of clinical proteomics is the clinical application of the findings. If this aim is ultimately not reached, the study has obviously failed reaching its primary objective. This does not necessarily render the study worthless, additional secondary goals like informing on disease mechanisms or molecular pathophysiology may still be reached and of relevance for the scientific community. At the same time, the mere publication of the results in a scientific journal, frequently a goal in the academic community, cannot be considered a success for a clinical proteomics project. Of even more relevance: if the goal of implementation is not pursued in a sound and credible way, then the study is in fact invalid from the beginning, and no efforts should be wasted on such invalid studies.

The data and reports currently available clearly indicate (although too often do not yet demonstrate) a potential major benefit for patients, depending on our ability to actually implement the findings as clinical tests or novel therapeutic drugs or regimens. It is consequently the responsibility of us, to do our best and deliver on the promises of the past, which are realistic, but obviously need to circumvent/eliminate the multiple obstacles on the way, many of which are not of a clinical or scientific nature. We owe it first and foremost to the patients to find ways dealing with these issues, so they can truly benefit from our clinical and scientific developments.

## Conflicts of Interest

Harald Mischak is the co‐founder and co‐owner of Mosaiques Diagnostics. The other authors declare no conflicts of interest.

## Data Availability

The author has nothing to report.
